# Capturing what matters: updating NICE methods guidance on measuring and valuing health

**DOI:** 10.1007/s11136-022-03101-6

**Published:** 2022-03-05

**Authors:** Dalia Dawoud, Alan Lamb, Alan Moore, Caroline Bregman, Ewa Rupniewska, Thomas Paling, Verena Wolfram, Rosemary E. S. Lovett, Ross Dent

**Affiliations:** 1 National Institute for Health and Care Excellence (NICE), Manchester, UK; 2 NHS England and NHS Improvement, Medicines Repurposing Program, London, UK

**Keywords:** Health technology assessment, Utilities, Health-related quality of life, Carer, Children, Age-adjustment

## Abstract

In July 2019, the National Institute for Health and Care Excellence (NICE) initiated a major review of its health technology evaluation methods to update its methods guide. This update has recently concluded with the publication of its health technology evaluation manual in January 2022. This paper reports the methods and findings of the review in relation to the recommended approach to use for the measurement and valuation of health-related quality of life (HRQoL) in submissions to NICE. Issues related to (i) the methods to use when NICE’s preferred measure (EQ-5D) is not appropriate or not available; (ii) adjusting health state utility values over time to account for age; (iii) measuring and valuing HRQoL in children and young people; and (iv) including carers’ QoL in economic evaluations were included in this review. This commentary summarises the methods used to undertake the review, its findings, and the changes to NICE methods that were proposed based on these findings. It also outlines topics where further research is needed before definitive methods guidance can be issued. The broad proposals described here were subject to a public consultation in 2020 and a further consultation on the updated methods guidance was completed in October 2021 before the publication of the manual in January 2022.

## Introduction

The National Institute for Health and Care Excellence (NICE) assesses the effectiveness and value for money of interventions and provides guidance to the UK National Health Service (NHS) on their use. To inform NICE guidance, evidence on how conditions and interventions affect people’s health-related quality of life (HRQoL) is crucial: it helps NICE committees assess clinical effectiveness, the impact of adverse effects, and cost effectiveness.

There are many ways of measuring HRQoL and converting that information into a value on the utility scale (where one is perfect health and zero is equivalent to being dead). To ensure consistency, NICE’s methods guide routinely specified a ‘reference case’. Companies are expected to submit evidence using reference-case methods but are permitted to submit alternative analyses, provided they explain why that is [1]. As an example, the reference case specifies the EQ-5D instrument; occasionally companies submit supplementary data using other measures.

Since NICE’s methods guide was last updated in 2013, academic research has progressed and the health and social care landscape has changed. Innovative interventions (such as personalised medicine and cell therapies) can be challenging to evaluate and there is demand for products to be made available more quickly, sometimes with a lower evidence base than was previously the case. To meet these and other challenges, NICE has just completed an update of its methods guide for health technology evaluation and published a new health technology evaluation methods and processes manual. This paper reports on one subsection of the wider update: the work and recommendations related to the measurement and valuation of HRQoL.

## Methods

A final specification of the work required to update this section was approved by a steering group convened to oversee the methods guide update in August 2019 [2]. This outlined the methodological issues that should be explored, which included the use of other measures of HRQoL than EQ-5D, adjusting health state utility values over time to account for age, measuring and valuing HRQoL in children and young people, carers’ HRQoL and its inclusion in economic evaluations and the choice of an EQ-5D-5L value set.

A ‘Task and Finish group’ was established to review the methods in the defined areas, assess the evidence base and the case for making changes. The group included NICE staff, representatives from charities and patient organisations, academia and the life sciences industry.

The general approach used was to conduct targeted reviews of the published literature, NICE Decision Support Unit (DSU) technical support documents (TSDs) and other key publications, with a focus on systematic reviews as the main publication type to consider, in each of the defined topic areas. Relevant NICE Technology Appraisals (TAs) and Highly Specialised Technologies (HST) guidance were also reviewed to supplement the literature reviews where needed. For some topics requiring more in-depth consideration, NICE DSU was commissioned to conduct full systematic reviews. Where assessment of the psychometric performance of patient-reported outcome measures (PROMs) was carried out this focussed on validity, reliability and responsiveness [3, 4]. Not all aspects that are relevant for PROMs are relevant for preference-based measures. For example, internal consistency reliability examines whether items within a measure are measuring the same construct, which is important for a PROM but not for a preference-based measure. The Task and Finish Group met 4 times to discuss the evidence and preliminary recommendations and agree whether there was a case for recommending a change to NICE methods in each of the areas examined. A second public consultation on the proposals has been completed and the final manual has been published in January 2022 [5].

### Findings

#### The use of HRQoL measures other than EQ-5D

##### When EQ-5D is not appropriate

The targeted literature review identified 4 publications from NICE DSU [6–9], and 4 relevant systematic reviews [10–13]. The most comprehensive publication was a review of reviews by Finch et al. (2018) that aimed to summarise the validity and responsiveness of 5 generic preference-based measures: EQ-5D, SF-6D, HUI3, AQoL and 15D (15 Dimensions), across a variety of disease areas [13].

The findings of our targeted review suggested that EQ-5D is appropriate for most conditions. There was evidence that EQ-5D may not be appropriate for hearing-related conditions [10]. For visual impairment, evidence suggested that EQ-5D did not perform well for age-related macular degeneration and diabetic retinopathy. Results were mixed in cataracts, whereas evidence supported its use in other eye conditions [7]. For mental health, conclusions on the appropriateness of EQ-5D varied from one condition to another with no problems identified for depression and anxiety, whereas evidence suggested that EQ-5D might have limitations for schizophrenia, personality disorders and alcohol dependency [14–17]. EQ-5D may also lack validity and/or responsiveness in HIV and dementia [18, 19]. Mixed evidence on EQ-5D appropriateness was found in multiple sclerosis [20].

We also reviewed published NICE appraisals and found that 7 TAs were identified where EQ-5D’s appropriateness may have been a concern including in hearing disorders, visual disorders and psychological disorders [21]. In these TAs, the committee preferred EQ-5D when it was presented together with other alternatives. The committee accepted other measures only when EQ-5D data were not available [21].

Additionally, we reviewed all published NICE HST evaluations completed by February 2020 (*n* = 12) and found that in half of the cases, EQ-5D data were collected in the trials or available from the literature [22]. Other generic measures, such as the SF-36 or HUI3, were used in a quarter of cases. Different methods were also used to obtain EQ-5D values, such as experts completing EQ-5D questionnaires to value vignettes or using mapping algorithms to derive EQ-5D values from another measure. In the evaluation documents, there were no records of data presented to demonstrate that EQ-5D is inappropriate (e.g. poor responsiveness or lack of validity). More frequently, the committee had concerns about the robustness of the methods used to derive the EQ-5D data [22].

Reviewing the published HST evaluations showed that vignettes are often used (33%), but the methods employed to value them varied, and raised concerns among the committee in some instances. Vignettes were more often scored by the clinicians than general public or people with the condition [22].

Overall, reviewing NICE appraisals and the published evidence suggests that the EQ-5D works well for most diseases and conditions. It was therefore recommended that EQ-5D should continue to be recommended as the preferred measure of HRQoL as stated in the NICE 2013 methods guide and that companies should present evidence of EQ-5D’s inappropriateness to justify the use of alternative measures [1]. This evidence should include an assessment of validity, responsiveness and reliability as outlined by NICE DSU.

However, it is acknowledged that for rare diseases, there may not be sufficient published literature to provide evidence that the EQ-5D does not perform well on psychometric measures. In such cases, a lack of content validity could be supported, by providing evidence that the EQ-5D lacks specific dimensions of health that are important to patients.

##### When EQ-5D data is not available

When evaluating rare diseases, or more generally, rare health states, EQ-5D data is often not available, or data obtained from clinical trials or from the literature may be sparse or of poor quality. The NICE 2013 methods guide did not specify what to do in these situations to populate an economic model. So, different approaches have been used to gather or generate utility values in past appraisals which has led to inconsistencies.

A recent review by the NICE DSU explored alternative methods for measuring and valuing health-related quality of life when insufficient EQ-5D data are available [23]. It suggested that using vignettes to generate EQ-5D utility data can be considered only if EQ-5D utility values cannot be sourced from the literature, utilities cannot be mapped, or a study to collect EQ-5D data cannot be conducted. It highlighted that, when completing EQ-5D for a vignette description, responses from people with the condition or the general population are preferable.

**Fig. 1:**
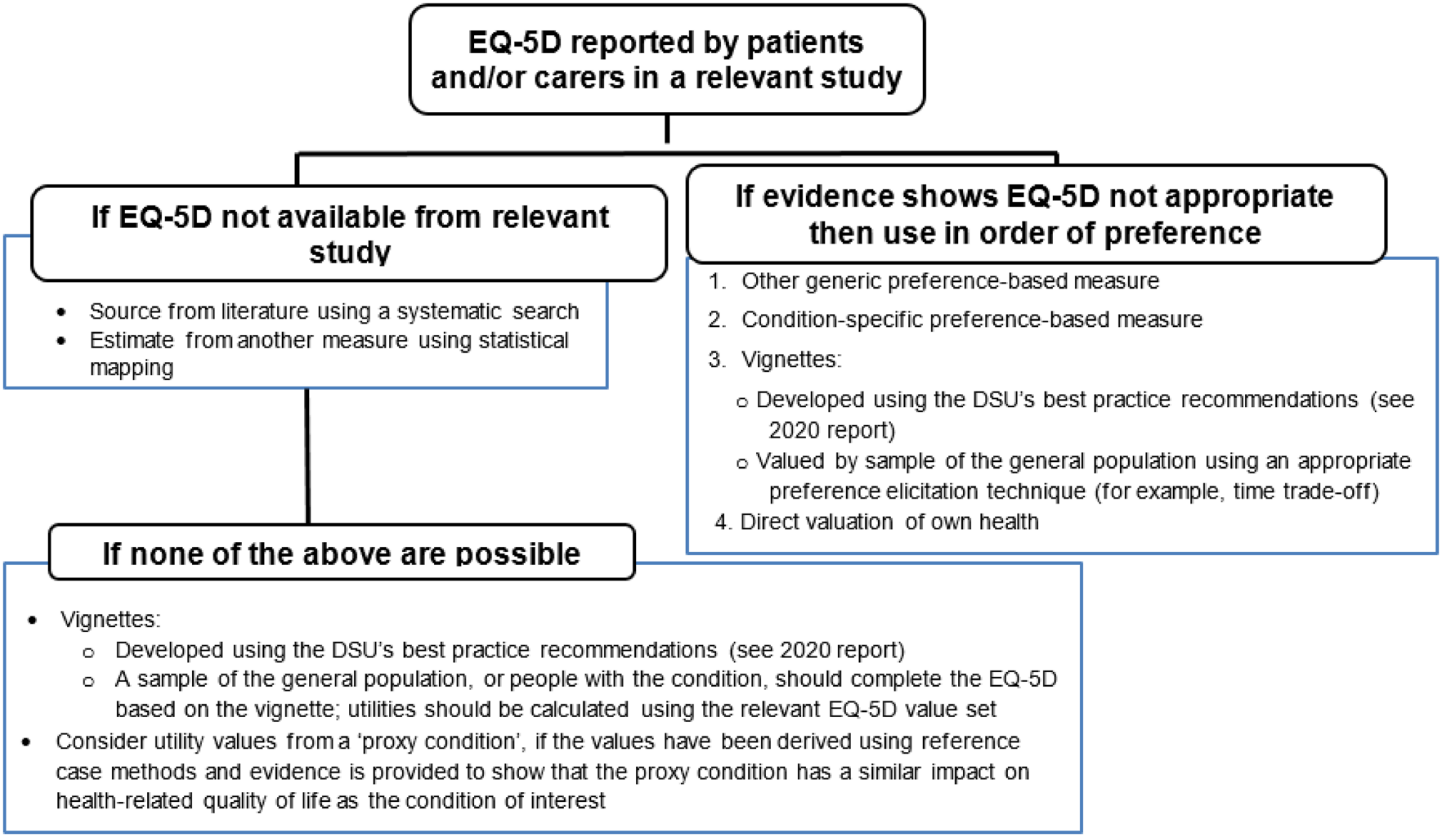
Hierarchy of preferred health-related quality of life methods [5]

The Task and Finish group proposed that the methods guide should include a figure (see Fig. 1) depicting preferred methods, and what alternative methods to use if EQ-5D data are not available or EQ-5D is inappropriate for a condition.

#### Adjusting health state utility values over time to account for age

A gradual decline in HRQoL has been observed as people get older, possibly due to an increasing number of comorbidities or because there may be a natural decline in mental and physical functioning with age [24, 25]. There is a consensus that, when extrapolating HRQoL data over long time horizons, it is appropriate to adjust them for this decline, analogous to adjusting the mortality rates. Adjusting health state utility values over time to account for age is recommended by ISPOR as best practice [26]. Adjustment ensures that economic models accurately capture the difference a technology can make to someone’s HRQoL, and that utility values do not exceed values expected for the general population at a given age.

The review of NICE case studies showed that adjusting utility values over time can make a difference to decision making, with ICERs increasing by 2.5% to 9.3% when age adjustment has been applied [27]. However, the review also showed that this adjustment was done inconsistently between appraisals and often methods used were not reported. Generally, the multiplicative approach (assuming a constant relative utility decrement) was used more often than the additive approach (assuming a constant absolute utility decrement), and EQ-5D general population distribution was usually sourced from either Ara and Brazier 2011 [25] or Kind et al. 1999 [24].

Exploratory analyses showed that multiplicative and additive methods produced similar adjusted utility values at higher baseline utility values. However, at lower baseline utility values, adjusted values using additive methods can be considerably lower than when using multiplicative method [27]. In specific circumstances, the additive approach can lead to utility values close to zero, or negative, which does not occur with the multiplicative approach. These very low or negative values are not realistic in most cases and lack face validity. A limitation of both methods is that they assume that the disutility multiplier or decrement related to a particular condition or event is constant over time. Furthermore, the exploratory analyses did not indicate that any age groups are disproportionately affected by adjusting utility values over time [27]. This is because although the decline in HRQoL is most pronounced at older ages, when extrapolating HRQoL over a lifetime horizon, younger cohorts will also reach these older ages.

Based on these findings, the Task and Finish group proposed that the methods guide should be updated to state that:

- If baseline utility values are extrapolated over long time horizons, they should be adjusted to reflect the decline in quality of life seen in the general population and to ensure that they do not exceed general population values at a given age.
- If this is not considered appropriate for a particular model, supporting rationale should be provided.
- A multiplicative approach is generally preferred, and the methods used for adjusting utility values should be clearly documented.

#### Children’s quality of life

NICE 2013 methods guide does not specify how to measure and value health-related quality of life in children and young people [1]. Consequently, past NICE evaluations showed wide variation in methods and widespread use of adult questionnaires [28, 29]. A recent review by the NICE DSU explored the psychometric properties of several preference-based measures used for children and young people [30, 31]. It found that the evidence is based on a relatively small number of studies across a range of countries, a range of different populations, using different study designs, different languages, different value sets and many different statistical techniques. Based on this, it was concluded that the academic literature is not mature enough to enable NICE to recommend specific health-related quality of life measure(s) and value set(s) for children and young people Therefore, more research is recommended in this area [5, 32].

The Task and Finish Group thus recommended that the updated methods guide should recommend using generic measure(s) that have good psychometric performance in the relevant age range(s). It acknowledged that not all paediatric questionnaires have value sets (and thus are difficult to include in an economic evaluation) but nonetheless they give valuable information about the impact of the condition and intervention on children and young people. It also proposed that companies should report whether questionnaire(s) were completed by children and young people themselves, adults with the condition, or proxies. If multiple data sources are available, companies should report which data were used in the economic model and their rationale.

#### Carer quality of life

Many people provide informal care to others such as partners or family members. The extent of this care, and the health impact on those who provide it, can vary substantially across disease areas. The NICE 2013 methods guide stated that “*all direct health effects, whether for patients, or when relevant, carers”* should be considered in an appraisal. However, there is no further guidance outlining a preferred approach when the case for including carer HRQoL is made [33].

A recent review by the NICE DSU found that only 12 out of 422 published TAs and HST guidance included health effects for carers [34]. It was more common in HST evaluations, perhaps due to more specific references to carer HRQoL in the HST interim methods and process guide, published in 2017, and the severity of conditions considered by the HST programme. There was a lack of good quality evidence to inform the relative impact of a new technology compared to current treatments on carer HRQoL. However, in most cases inclusion of carer HRQoL significantly impacted on cost-effectiveness results. Therefore, there is a need to ensure it is captured appropriately and robustly. Nevertheless, there is no consensus of academic opinion on appropriate methods and further research is needed on the technical issues of the inclusion of carer HRQoL in economic models.

The Task and Finish group agreed there are some occasions when it is relevant to include carer HRQoL in appraisals. It was, thus, proposed that including a set of minimum standards may help to ensure that submitted evidence relating to carer HRQoL is more robust and could result in less variation in methods across appraisals. The Task and Finish group has drafted an initial set of minimum standards but considered that these should be informed by input from a range of stakeholders because many of the items require normative judgments [27]. For example, whose HRQoL should be considered—just the main carer(s) or wider family members? The final manual therefore states that evidence should be provided to show that the condition is associated with a substantial impact on carer HRQoL, without setting minimum standards at this time [5].

#### EQ-5D-5L value set and mapping between different versions of the EQ-5D

The EQ-5D-5L is a version of the EQ-5D where respondents can rate their health using 5 levels of severity. The EQ-5D-5L was designed to be more sensitive than the EQ-5D-3L, where respondents can choose from 3 levels of severity. NICE has previously published a position statement on the use of the EQ-5D-5L, which states that the EQ-5D-5L questionnaire may be used to collect quality-of-life data but that the UK EQ-5D-3L value set should be used [35]. EuroQol has commissioned a new 5L valuation study for England using an updated international standard protocol [35].

Mapping approaches are required to use the EQ-5D-3L value set with response data from the EQ-5D-5L questionnaire. The methods and data sets available to map data from the EQ-5D-5L to the EQ-5D-3L were reviewed. The review identified that a new tool developed by NICE’s Decision Support Unit (DSU) has additional functionality compared with the previously preferred tool (by van Hout et al. 2012 [36]), notably the ability to map directly from utility scores if individual EQ-5D response data are not available, [37] and is also informed by a larger dataset [37].

The Task and Finish Group proposed that the updated methods guide should incorporate the 2019 NICE position statement on the use of the EQ-5D-5L value set for England [35]. It was also proposed that the preferred method for mapping from EQ-5D-5L to EQ-5D-3L should be using the DSU’s tool, based on the dataset collected by Hernández Alava and colleagues (the ‘EEPRU’ dataset) [37].

### Future research priorities

The Task and Finish Group identified the research priority areas to be those relating to children’s and carers’ HRQoL. For children’s HRQoL, substantial research is needed to explore the appropriateness of different measures for use in children and young people, and to identify appropriate valuation methods. Of particular interest is research examining the content validity of different measures, or directly comparing psychometric performance in large head-to-head studies. Choosing a valuation approach requires further research into how the choice of population, perspective and valuation method impact valuation results. NICE is actively involved in cross-agency collaborations and supporting academic research in these areas.

For carer HRQoL, further research is needed to clarify when and how carer quality of life should be included in evaluations. This research must consider the effects of displacement and opportunity cost, that is, healthcare displaced by a new technology would itself have effects on carers and family members, which must be taken into account. Based on the above, the Task and Finish Group recommended prioritising reviewing and reconsidering NICE methods in these areas as the ongoing research progresses.

## Conclusions

Adequate measurement and reporting of HRQoL in NICE submissions is a key step in ensuring that the impact of a technology on patients’ and carer’s lives is reflected accurately in economic evaluations. The outlined changes to NICE methods are based on the best available and most up-to-date evidence. Adopting these changes should improve practice and ensure a health technology’s impact on HRQoL is accurately reflected in NICE evaluations.

## Data Availability

Material and data available from https://www.nice.org.uk/about/what-we-do/our-programmes/nice-guidance/chte-methods-consultation
